# Branching Pattern of the Cerebral Arterial Tree

**DOI:** 10.1002/ar.23994

**Published:** 2018-12-05

**Authors:** Jasper H. G. Helthuis, Tristan P. C. van Doormaal, Berend Hillen, Ronald L. A. W. Bleys, Anita A. Harteveld, Jeroen Hendrikse, Annette van der Toorn, Mariana Brozici, Jaco J. M. Zwanenburg, Albert van der Zwan

**Affiliations:** ^1^ Department of Neurosurgery University Medical Center Utrecht Utrecht The Netherlands; ^2^ Brain Technology Institute Utrecht The Netherlands; ^3^ Departent of Anatomy Radboud University Medical Center Nijmegen The Netherlands; ^4^ Department of Anatomy University Medical Center Utrecht Utrecht The Netherlands; ^5^ Department of Radiology University Medical Center Utrecht Utrecht The Netherlands; ^6^ Department of Pulmonology Heilig Hart Ziekenhuis Mol Belgium

**Keywords:** anatomical research, branching patterns, cerebral arterial circulation, high resolution MRI, minimum work

## Abstract

Quantitative data on branching patterns of the human cerebral arterial tree are lacking in the 1.0–0.1 mm radius range. We aimed to collect quantitative data in this range, and to study if the cerebral artery tree complies with the principle of minimal work (Law of Murray).

To enable easy quantification of branching patterns a semi‐automatic method was employed to measure 1,294 bifurcations and 2,031 segments on 7 T‐MRI scans of two corrosion casts embedded in a gel. Additionally, to measure segments with a radius smaller than 0.1 mm, 9.4 T‐MRI was used on a small cast section to characterize 1,147 bifurcations and 1,150 segments. Besides MRI, traditional methods were employed. Seven hundred thirty‐three bifurcations were manually measured on a corrosion cast and 1,808 bifurcations and 1,799 segment lengths were manually measured on a fresh dissected cerebral arterial tree. Data showed a large variation in branching pattern parameters (asymmetry‐ratio, area‐ratio, length‐radius‐ratio, tapering). Part of the variation may be explained by the variation in measurement techniques, number of measurements and location of measurement in the vascular tree.

This study confirms that the cerebral arterial tree complies with the principle of minimum work. These data are essential in the future development of more accurate mathematical blood flow models. Anat Rec, 302:1434–1446, 2019. © 2018 The Authors. *The Anatomical Record* published by Wiley Periodicals, Inc. on behalf of American Association of Anatomists.

Blood flow modeling could aid in better understanding of hemodynamics in many cerebrovascular diseases such as aneurysms, vascular dementia, moyamoya vasculopathy, and stroke. However, for cerebrovascular blood flow modeling to be successful, clear morphological knowledge of the cerebral arterial tree is needed (Hirsch *et al.,* 2012). This tree spans from the circle of Willis, which is known to be highly variable, up to the capillary network (Alpers *et al.,* 1959; Hillen, 1986; Hartkamp *et al.,* 1999). The main branches of the circle of Willis each form a large arterial tree, supplying different and variable vascular territories of the brain (van der Zwan and Hillen, 1991; van der Zwan *et al.,* 1992).

Morphological data on vascular trees are traditionally expressed by branching patterns described by mathematical relations between parent and daughter arteries at bifurcations as well as diameters and lengths of intermediate vessel segments (Koike *et al.,* 1986; Zamir, 1999).

In addition, it is generally accepted that the morphology of the cerebrovascular tree follows the principle of minimum work as described by Murray (1926a). This principle is expressed by the equation $r_{0}^{3}=r_{1}^{3}+r_{2}^{3}$, where according to convention *r*_*0*_ is considered to be the radius of the parent artery and *r*_*1*_ and *r*_*2*_ of both daughter arteries (Murray, 1926b; Sherman, 1981; Zamir, 1999). Morphological data confirming that the cerebral arterial tree adheres to this principle of minimum work are, therefore, essential and we believe this could aid in optimization of models for the human vasculature and ease hemodynamic flow modeling (Gabryś *et al.,* 2006).

In the past, data of branching patterns of an arterial tree were gathered using manual measurements on *ex vivo* and *in vivo* specimens (Koike *et al.,* 1986; Horsfield and Woldenberg, 1989; Papageorgiou *et al.,* 1990; MacLean *et al.,* 1992; Wang *et al.,* 1992; Rossitti and Löfgren, 1993; Zamir, 1999; Mittal *et al.,* 2005; Cassot *et al.,* 2006). More modern techniques utilize newer three dimensional (3D) imaging techniques such as computed tomography angiography (CTA) and magnetic resonance imaging (MRI) for *in vivo* capturing of larger arteries and micro‐computed tomography (micro‐CT) and confocal laser microscopy for capturing highly detailed *in vivo* and *ex vivo* vascular networks (Cassot *et al.,* 2006; Nordsletten *et al.,* 2006; Beare *et al.,* 2011).

To our knowledge, information on the branching pattern of the human intracranial cerebral arterial tree is still limited. Also information is limited on whether the human cerebral arterial tree complies to the principle of minimum work as described by Murray (Murray, 1926b; Suwa *et al.,* 1963; Rossitti and Löfgren, 1993; Ingebrigtsen *et al.,* 2004; Cassot *et al.,* 2009; Mut *et al.,* 2014). Most studies performed measurements on arteries larger than 1 mm (Rossitti and Löfgren, 1993; Ingebrigtsen *et al.,* 2004) whereas others studied only the microvasculature (Cassot *et al.,* 2006; Cassot *et al.,* 2010). Data on vessels with intermediate sizes (30 μm–1 mm) are scarce.

High resolution MRI scanning of corrosion casts has the potential to increase our knowledge on morphology in the smaller segments of the vascular tree (Helthuis *et al.,* 2018). The current study aims to add quantitative data on the branching pattern of the human cerebral arterial tree in the missing range between larger cerebral arteries and the capillary network by applying this new MRI based method using 7 T and 9.4 T MRI. Because MRI based methods are still relatively new, we also performed manual measurements on corrosion cast and dissection that will be presented along the MRI data as reference. This data could be used in future models of the cerebral arterial tree (Hirsch *et al.,* 2012). The data of the branching patterns are used to assess whether the human cerebral tree keeps the principle of minimum work as defined by the Murray's law.

## MATERIALS AND METHODS

### General Study Design

Four measurement techniques were used. The first two techniques were earlier described 7 T MRI and 9.4 T MRI technique to quantify a large number of vessels in radius range of 0.1–1.0 mm and 30–200 μm, respectively (Helthuis *et al*., 2018). Third, manual measurements on a pressurized vascular cast were performed. Finally, vessel diameters of a freshly dissected human arterial tree were measured.

Brains used in the current study were derived from bodies that were donated to the Department of Anatomy. From these persons written informed consent was obtained during life that allowed the use of their entire bodies for educational and research purposes. As this research complies with the given written informed consent, in accordance with the Dutch law on dead bodies, no further IRB approval was required.

### Casts

#### Preparation

**Figure 1:**
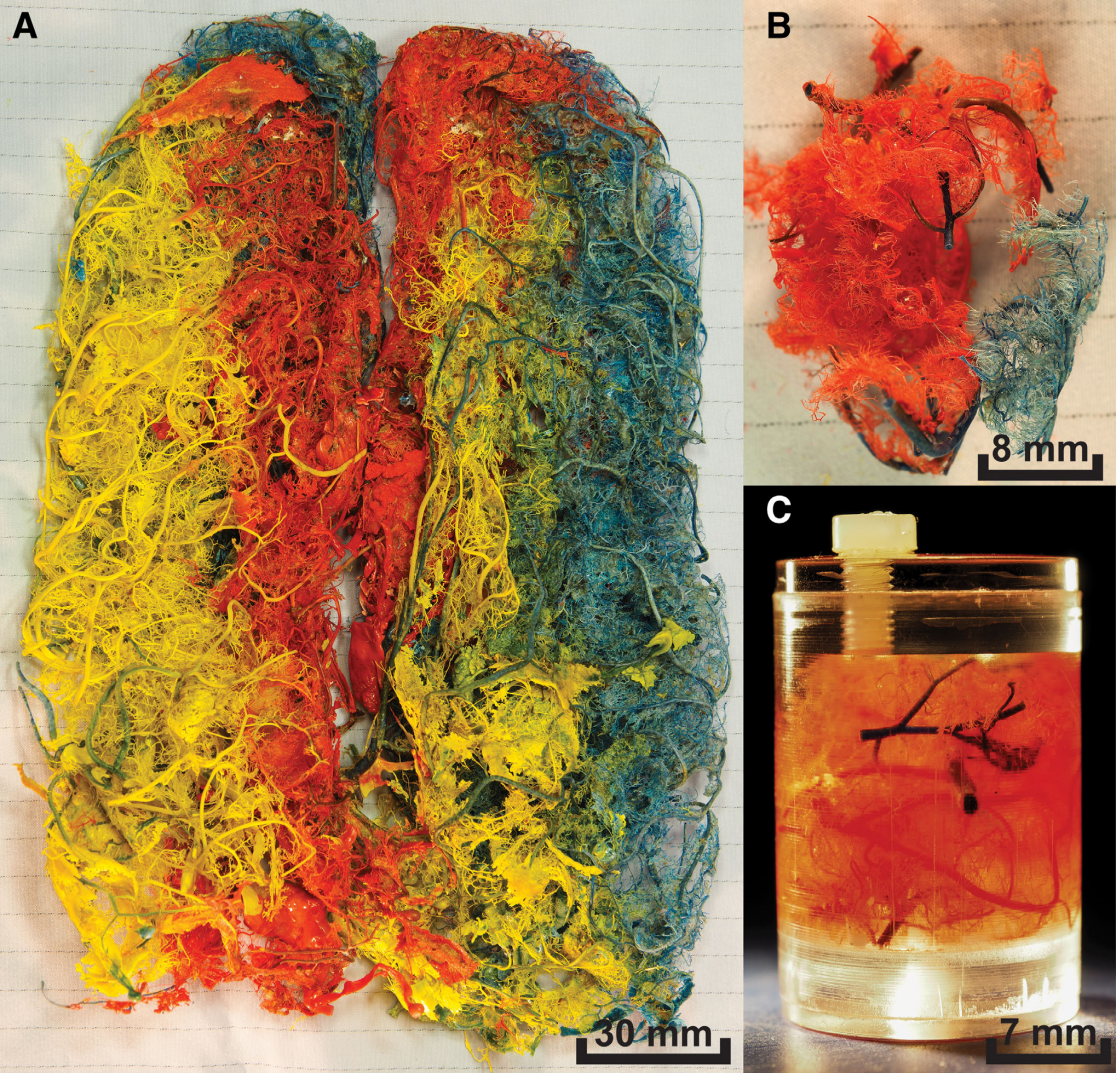
Examples of the casts: **(A)** Photograph of one of the plastic casts of the full cerebral arterial circulation, as was used in the 7 T MRI. Different color pigments where used for the six major cerebral arteries. **(B)** Photograph of a small section of the casts as was used in the 9.4 T MRI. **(C)** Photograph of the same small section of casts for the 9.4 T MRI placed in the gadolinium‐gelatine solution in a Perspex container.

For 7 T MRI two full casts were produced (one male, 50 years and one female, 78 years). For 9.4 T RMI a third cast (female, 57 years at 9.4 T MRI) was produced from which only a small section was used (Fig. 1A,B). For manual measurements a fourth full cast (female, 89 years) was produced and cut in smaller segments. None of the four used brains showed signs of pathology after harvesting and all were fully intact. The casts were created by the injection of a mixture of Araldite F/hardener HY 2967/dilutioner DY 026 SP through cannulas in the six major cerebral arteries of the brain under controlled pressure. The brains were subsequently corroded. This procedure was previously described in detail (Van Der Zwan and Hillen, 1990).

#### MRI (7 T and 9.4 T)

##### MRI scanning and preparation

**Figure 2:**
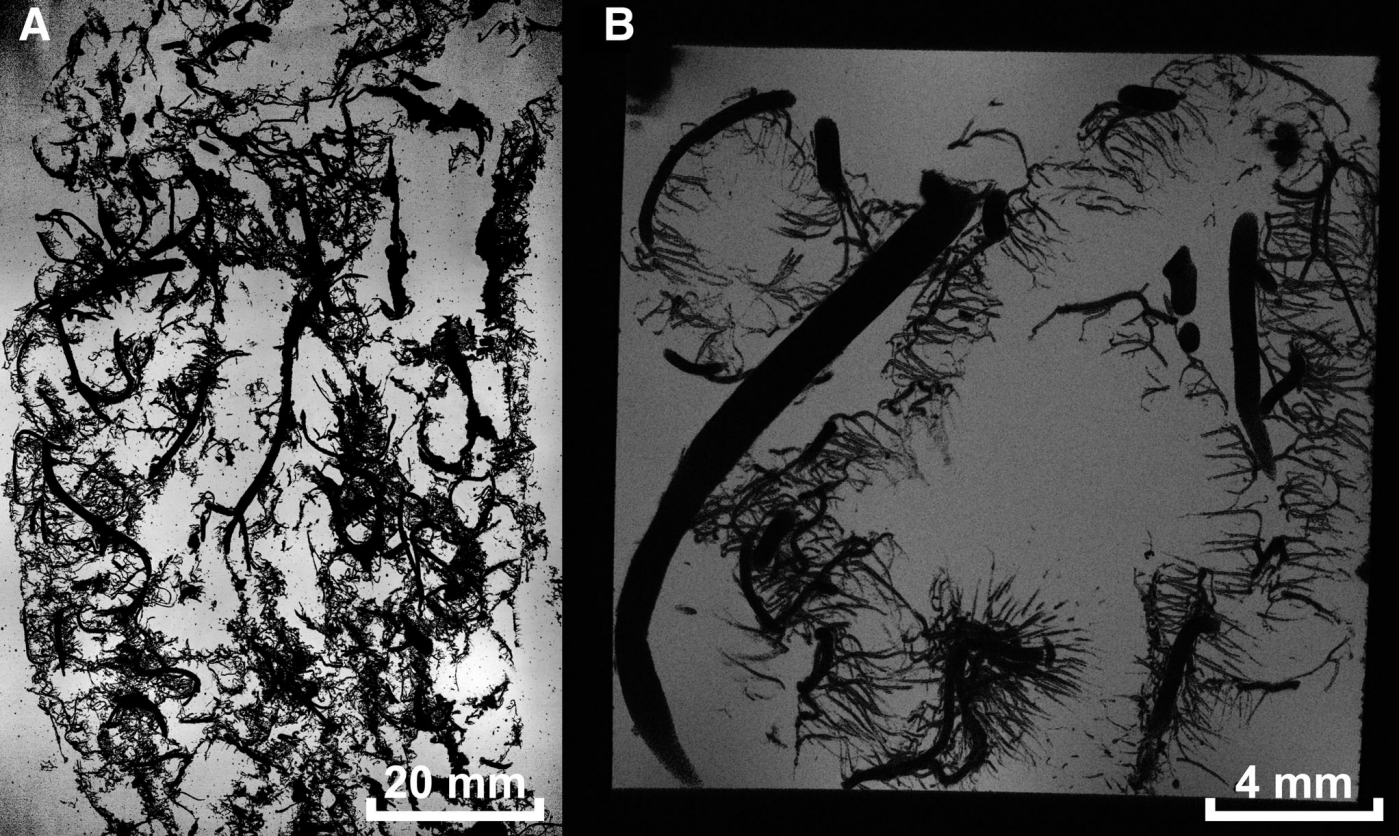
Example MRI data. **(A)** Minimum intensity projection of 20 slices (1.95 mm total thickness) of 7 T MRI data. **(B)** Minimum intensity projection of 100 slices (1.50 mm total thickness) of 9.4 T MRI data.

MRI protocols and postprocessing were previously described (Helthuis *et al*., 2018). In summary, to prepare for MRI scanning two complete casts of the cerebral circulation (7 T MRI) and a small section of another cast were embedded in solution of gelatine (14%, powder, Sigma‐Aldrich, Saint Louis, MO) and a gadolinium‐containing contrast agent (2.8*10^‐3^ mL contrast agent/mL water; Gadobutrol, Gadovist 1.0 mmol/mL, Bayer Schering Pharma, Newbury, UK) in water, and subsequently placed in a custom made PCV container (7 T MRI) or Perspex container (9.4 T MRI; Fig. 1C). Next, 7 T MRI was performed on a whole‐body system (Philips Healthcare, Cleveland, OH) with a 60 cm bore diameter. The acquired voxel size was of 0.1 × 0.1 × 0.1mm^3^. 9.4 T MRI was performed on a 9.4 T/21 cm MR system (Varian Inc. Palo Alto, CA) with an acquired voxel size of 30 × 30 × 30 μm^3^.(see Fig. 2 for example of MRI data).

To create the 3D reconstruction, a semi‐automatic segmentation method was used in which the user manually selects locations along the arterial tree. Between these locations centrelines were traced. These centrelines consist of many separate connecting nodes at which for each node the radius was calculated using a full‐with‐at‐halve maximum method.

##### Calculation of radii and lengths

The arterial trees acquired by MRI scanning were split in separate segments between bifurcations. For each segment length, proximal radius and distal radius were calculated using a custom MATLAB code (version R2014a (8.3.0.532), The Mathworks, Inc). Each segment consists of many separate nodes with respective radii. To calculate a representative radius at the proximal and distal side of the segments a method had to be devised. The first and last quartiles of nodes of each segment were not used as it is known that just after and before a bifurcation the radius decreases and does not portraits the actual radius of a segment (Hardy‐Stashin *et al.,* 1980; MacLean *et al.,* 1992). Using the radius of the nodes of the middle quartiles and their Euclidian distance from the start of the first node, a linear regression analysis was performed. Based on this linear regression analysis the proximal and distal radius were calculated. Length was calculated as the sum of all Euclidean distances between all nodes forming one segment.

Segments with a proximal diameter smaller than two voxel size and bifurcations with parent or daughter arteries with a diameter smaller than two voxel size were excluded from further analysis as two voxels are required to more accurately calculate diameters (Bouvy *et al.,* 2014). Finally, this resulted in a database with length, radii, and connective information for each segment in the arterial tree based on MRI data. Data of both 7 T MRI casts were combined for ease of further calculations.

#### Manual measurements

Subsequently one cast was divided in smaller sections. On each section the diameters of parent and both daughter arteries were measured for every accessible bifurcation using a digital caliper under a microscope (OPMI‐1, Carl Zeiss Meditec Inc., Dublin, CA, magnification 10‐40x). Manual measurement of lengths on our casts was technically not possible.

### Dissection

#### Brain preparation

A fresh human cadaver (male, 71 years) was decapitated within 24 hr postmortem. Both common carotid arteries and vertebral arteries were cannulated and flushed with laundry detergent dissolved in water at room temperature (6–8 g/L. OMO Professional, Unilever N.V., Rotterdam, The Netherlands) until clear fluid returned through the internal jugular veins. Next a mixture of water, 20% gelatine, and a 5% red gouache paint (Ecola, Royal Talens, Apeldoorn, The Netherlands), which would aid in subsequent dissection, was injected until the mixture clearly returned through the venous system. To prevent the gelatine paint mixture from leaking out of the vasculature, clamps were placed on the carotid and vertebral arteries, jugular veins, and other collateral cervical veins and arteries showing leakage of fluid. Finally, the head was submerged in cold (5° Celsius) laundry detergent solution (6–8 g/L) and placed overnight in the refrigerator at 5° Celsius for gelatine to solidify.

#### Dissection method

The brain was obtained by cutting its connecting structures to the skull base after removal of the skull cap. Next, the cerebellum was removed from the brain by a cut through the pons. The circle of Willis was identified. The six major cerebral arteries were cut at the level of the circle of Willis and their respective arterial tree dissected to the level of arteries with a diameter of roughly 200–300 μm. Dissection was performed using an operating microscope (OPMI 6‐CFC, Carl Zeiss Meditec Inc., Dublin, CA, magnification 10‐40x). Dissected arterial trees were stored at 5° Celsius in a mixture of laundry detergent and formaldehyde (6–8 g/L OMO professional, 0.3%–0.5% Formaldehyde).

#### Measurement method

**Figure 3:**
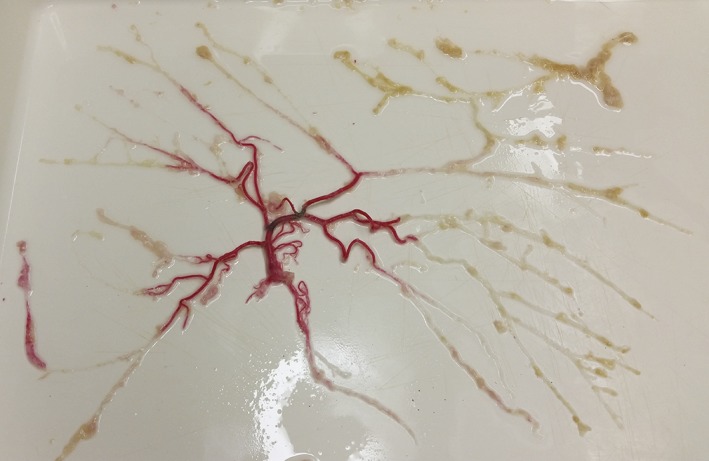
Dissected cerebral arterial tree of the right middle cerebral artery spread out on the table. Arteries are still partially filled with the gelatine‐red paint solution.

First the gelatine paint solution was removed from the arterial tree by spreading the arterial tree (Fig. 3) on a flat surface and carefully manipulating the gelatine in the direction of greater diameter arteries. Next, the diameters of each bifurcation and lengths between bifurcations were measured.

Measurements of radius were done as described in more detail by Hillen (1986). In summary, the arteries were flattened on a flat surface under a clear glass microscope slide. The width of the flattened vessel was measured on top of this microscope slide using a digital caliper. Next, the vessel wall thickness was determined on the same location as where the width was measured by flattening the artery between de tips of the digital caliper just until the tips started to indent the arterial wall. Using this width and vessel wall thickness the radius was calculated according to

$$\text{radius}=\frac{\text{width}-2\times \text{vesselwall}}{\pi }$$

For measurement of lengths, segments between bifurcations were carefully stretched until straight and subsequently measured using a digital caliper.

All measurements were performed under the same operating microscope aforementioned. Measurements of radius and length were all performed twice and averaged to mitigate measurement errors.

### Data Analysis

#### Statistical analysis and definitions

Definitions for different parameters in the branching patterns of the cerebral arterial tree that were of interest to the current study are displayed in Table 1. The lenght‐to‐radius ratio (LR) and tapering (T) were calculated from dissection‐ and MRI‐data only as measurement of length was not feasible by manual cast measurements. From the data using MRI, the branching pattern parameters were calculated using MATLAB. From the direct measurements on cast and dissection, these calculations were performed using Microsoft Excel 2013 (version 15.0.4745.1000, Microsoft Corp.)

**Table 1:** Definitions

Definition	Description
Bifurcation/trifurcation	Location where an artery splits into two/three separate arteries
Segment	Piece of artery between two bifurcations
Parent artery	Inlet artery of a bifurcation. r_0_ represents the radius of the parent artery
Daughter artery	Outflow artery of a bifurcation. *r*_1_ and *r*_2_ represent the radii of the two daughter arteries, where according to convention *r*_1_ is the largest radius
Proximal artery radius	Radius at the proximal (upstream) side of a segment between two bifurcations
Area ratio (AR)	The ratio between the cross‐sectional area of the parent artery and the sum of the cross‐sectional area of all daughter arteries. This is for bifurcations calculated as: $\mathrm{AR}=\frac{\pi r_{1}^{2}+\pi r_{2}^{2}}{\pi r_{0}^{2}}\;$
Asymmetry ratio (AS)	The ratio between the cross‐sectional areas of the smallest daughter artery (*πr*_*2*_^*2*^) and the the largest daughter artery (*πr*_*1*_^*2*^ *)*. This ratio can only be calculated for bifurcations and not for junctions with more than two daughter arteries such as trifurcations: $\mathrm{AS}=\frac{\pi r_{2}^{2}}{\pi r_{1}^{2}}$
Length‐to‐radius ratio (LR)	The LR was defined as the length (*L)* of an arterial segment divided by its radius at the proximal end (*r*_proximal_) of this segment. $\mathrm{LR}=L/r_{\text{proximal}}$
Tapering (*T*)	The ratio between the radius at the distal end (*r*_distal_ *)* of a segment and the radius at the proximal end (*r*_proximal_ *)* of a segment. T=rdistalrproximal
Principle of minimum work	according to Murray(Murray, 1926b). Defined as: $r_{0}^{3}=r_{1}^{3}+r_{2}^{3}$ For analysis whether a bifurcation complies with this principle this formula was rewritten as: $\;\frac{r_{1}^{3}+r_{2}^{3}}{r_{0}^{3}}=n$ If *n* equals 1 a respective bifurcation complies with the principle of minimum work.

A Kolmogorov–Smirnov (KS) test was used to check normal distribution. In case the data was not normally distributed, the median, interquartile range (IQR), and range of measurements were reported, and histograms were made.

Linear regression analysis was performed to analyze the area‐ratio (AR) and the principle of minimum work. For (a) AR: regression was performed between the squared radius of the parent artery and the sum of the squared radii of all daughter arteries. The slope of this regression analysis would be the AR. (b) For the principle of minimum work: regression was performed between the cubed parent artery radius and the sum of cubed radii of all daughter arteries, where a slope of equal to one would mean the overall cerebral tree complies to the principle of minimum work. The robust regression and outlier removal (ROUT) as described by Motulsky and Brown (2006) was implemented in GraphPad Prism (version 6.01, GraphPad Software, Inc. La Jolla, CA), and used with a Q value of 1% to select and remove outliers. No outliers were removed from data on asymmetry ratio (AS) due to the fact that these values are limited in a range between 0 and 1. All statistical analyses were performed using GraphPad Prism. The datasets generated during and/or analyzed during the current study and MATLAB scripts are available from the corresponding author on reasonable request.

## RESULTS

### Lengths and Radii of Segments

**Figure 4:**
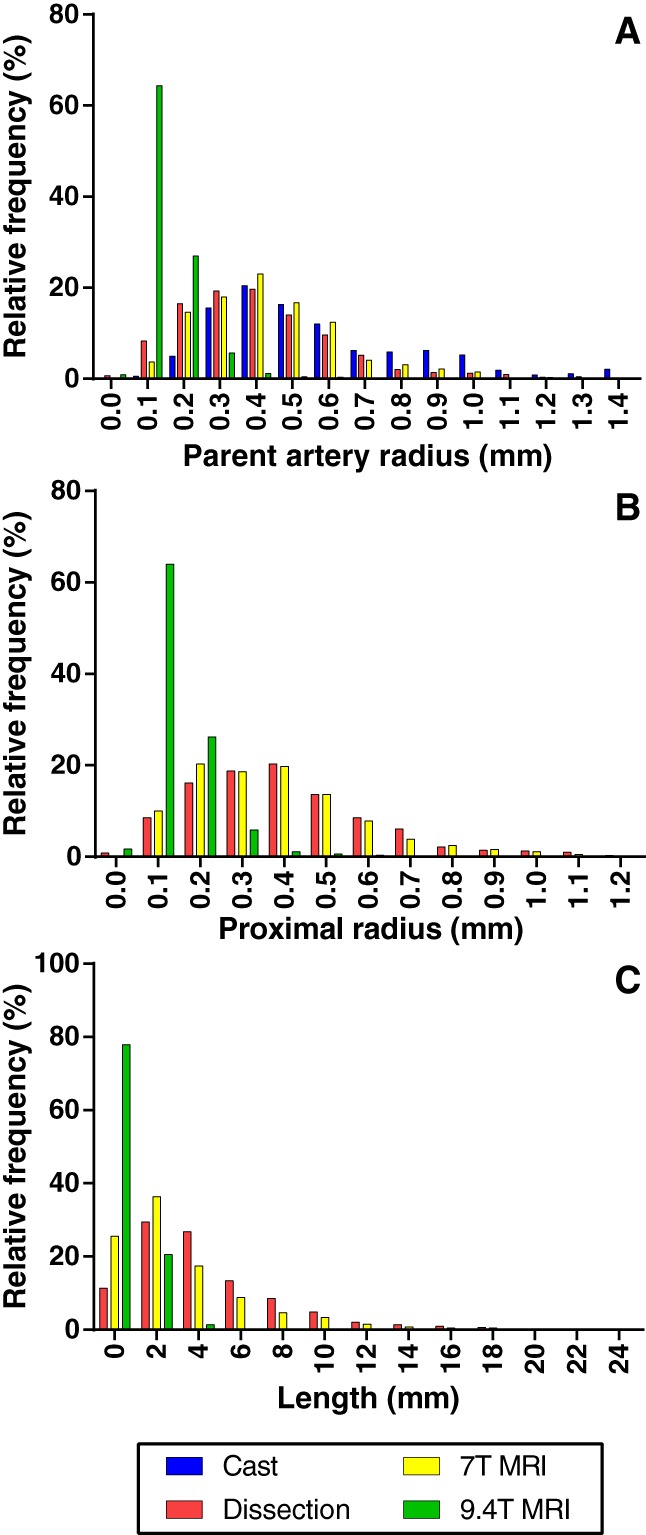
Relative frequencies in percentage (%) of *top*: parent artery radius in millimeters (mm) for bifurcations, *middle*: proximal radius in mm for segments, *bottom*: Length for segments in mm.

Table 2 shows the data on parameters. None of the parameters was normally distributed. A total of 1,294, 1,147, 733, and 1,808 bifurcations were available for analysis for 7 T MRI, 9.4 T MRI, manual cast and dissection data, respectively. A total of 2,031, 1,150, and 1,799 segments were available for analysis of the 7 T MRI, 9.4 T MRI, and dissection data, respectively. Figure 4 shows the distribution of the parent artery radius (Fig. 4 top), the proximal radius (Fig. 4 middle), and length (Fig. 4 bottom).

**Table 2:** General results. Radii are reported as median radius in millimeters (mm) with interquartile range (IQR) and range: median (IQR, range)

Source	Bifurcations/segments (n)	Parent artery radius (mm)	Proximal artery radius (mm)	Length (mm)
Cast	733	0.50 (0.35, 0.09–1.61)		
Dissection	1,808/1,799	0.37 (0.27, 0.03–1.59)	0.38 (0.27, 0.02–1.60)	3.60 (4.13, 0.13–35.68)
7 T MRI	1,294/2,031	0.41 (0.24, 0.10–1.41)	0.36 (0.26, 0.10–1.50)	2.20 (3.40, 0.00–41.53)
9.4 T MRI	1,147/1,150	0.12 (0.08, 0.04–0.67)	0.13 (0.08, 0.03–0.69)	0.49 (0.67, 0.00–5.68)

Between the different methods used to acquire data, there was a difference in distribution of included arterial diameters (Table 2 and Fig. 4 top and middle). Data from manual measurement on casts included larger arteries (median 0.5 mm range: 0.09–1.61) compared to dissection and 7 T MRI data (median 0.41 mm range: 0.10–1.41). 9.4 T MRI data included only small arteries up to the pre‐capillary network (median 0.12 mm range: 0.04–0.67).

### Branching Patterns

**Figure 5:**
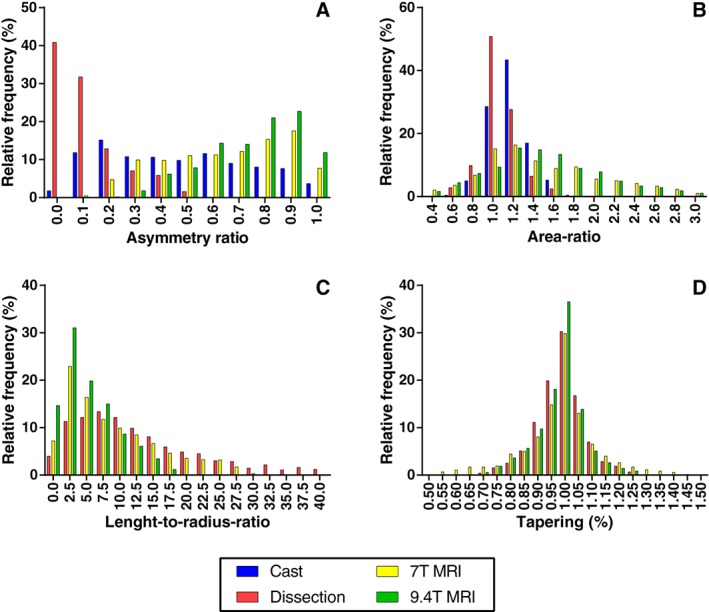
Relative frequencies of **(A)** asymmetry ratio, **(B)** area ratio, **(C)** length‐to‐radius‐ratio, **(D)** tapering.

**Figure 6:**
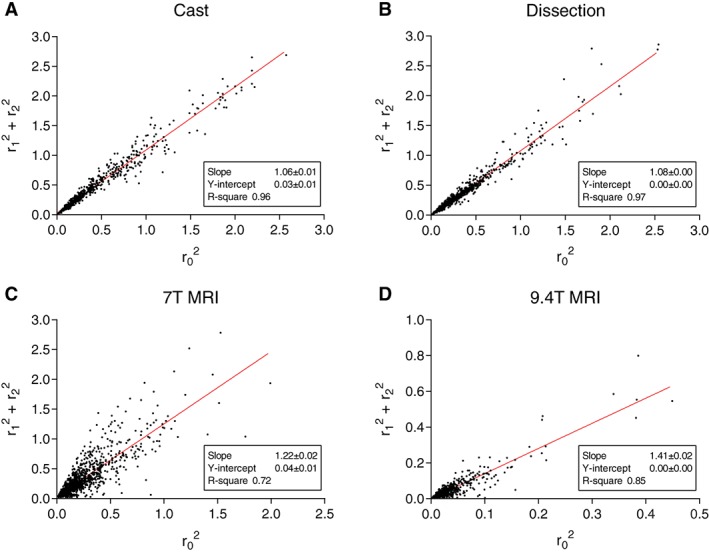
Area ratio. Scatterplots of squared parent artery radius (*r*_0_) versus sum of squared radii (*r*_1_, *r*_2_) of all daughter arteries for **(A)** cast, **(B)** dissection, **(C)** 7 T MRI, and **(D)** 9.4 T MRI.

**Figure 7:**
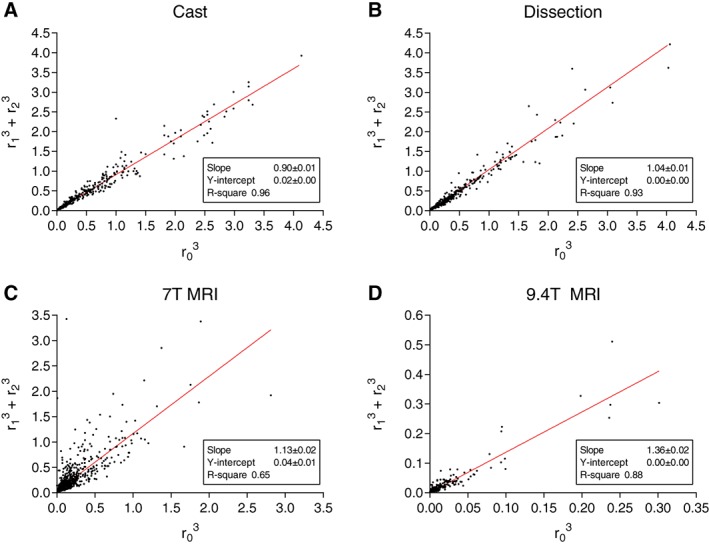
Principle of minimum work according to Murray. Scatterplots of cubed parent artery radius (r_0_) versus sum of cubed radii (*r*_1_, *r*_2_) of all daughter arteries for **(A)** cast, **(B)** dissection, **(C)** 7 T MRI, and **(D)** 9.4 T MRI.

Parameters characterizing the branching patterns are summarized in Table 3 and histograms in Figure 5. None of the resulting branching patterns were normally distributed. All branching patterns showed a broad distribution (Fig. 5). For data obtained from the MRI measurements, this spread in branching patterns seemed to be larger for the AR. The median AS turned out to be larger in MRI data then from manual measurement results. Linear regression analyses of the AR are shown in Figure 6. Linear regression analyses for the principle of minimum work according to the Law of Murray are shown in Figure 7. The AR showed a larger IQR in the two casts that were used for MRI, compared to the AR measured in the cast that was used for manual measurements (Table 3). Linear regression analysis (Fig. 6) showed a larger slope for MRI data for AR. LR data showed a lower median value for MRI data then for dissection data. Median values for tapering were 0.99 for both MRI techniques and dissection (Table 3). Regression analysis (Fig. 7) on dissection data shows a slope close to one (1.04 ± 0.01, *R* ^2^ = 0.97, *P* < 0.0001) confirming the principle of minimum work while the slope for manual measurements on the casts is smaller than one with a value of 0.9 ± 0.01 (*R* ^2^ = 0.96, *P* < 0.0001) and for 7 T MRI and 9.4 T MRI are larger than one with slopes of 1.13 ± 0.04 (*R* ^2^ = 0.73, *P* < 0.0001) and 1.36 ± 0.02 (*R* ^2^ = 0.85, *P* < 0.0001), respectively.

**Table 3:** Branching patterns of manual measurements on cast and dissected arteries and measurements using 7 T MRI and 9.4 T MRI

	AR	AS	LR	Tapering
Cast	1.16 (725, 0.22, 0.57–1.81)	0.44 (733, 0.47, 0.00–1.00)	NA	NA
Dissection	1.05 (1,733, 0.19, 0.51–1.61)	0.09 (1,808, 0.26, 0.00–1.00)	10.49 (1,681, 12.17, 0.22–42.38)	0.99 (1,745, 0.09, 0.71–1.27)
7 T MRI	1.38 (1,252, 0.84, 0.07–3.71)	0.68 (1,293, 0.40, 0.11–1.00),	6.82 (1,854, 10.03, 0.00–29.58),	0.99 (1,910, 0.11, 0.55–1.43),
9.4 T MRI	1.45 (1,123, 0.77, 0.05–3.63)	0.78 (1,146, 0.27, 0.17–1.00).	4.24 (1,098, 5.83, 0.00–17.45)	0.99 (1,065, 0.08, 0.70–1.27)

Values are all median (number without outliers, interquartile range, range). NA = not applicable, AR = area ratio, AS = asymmetry ratio, LR = length‐to‐radius ratio.

## DISCUSSION

To the best of our knowledge, this is the first study which shows branching patterns in the radius range between larger cerebral arteries (>1 mm) and the microvascular network (<100 μm). In the current study both 7 T MRI and 9.4 T MRI techniques were used. These techniques allowed for faster quantification than when done using manual measurement techniques and allowed characterization of a larger sample size and range than when solely a high resolution technique like micro‐CT, confocal microscopy, stained histology slices or 9.4 T MRI scanning would have been used (Motti *et al.,* 1986; MacLean *et al.,* 1992; Cassot *et al.,* 2006; Dickie *et al.,* 2006; Dutly *et al.,* 2006; Heinzer *et al.,* 2006; Nordsletten *et al.,* 2006; Lauwers *et al.,* 2008; Reichold *et al.,* 2009; Tsai *et al.,* 2009). Manual measurements on cast and dissected arteries were also performed to enable comparison to traditional techniques as MRI and reconstructive techniques are still relatively new in characterization of arterial branching (Hirsch *et al.,* 2012).

The data generated might be valuable for use in modeling of the cerebral arterial tree, which could improve understanding of different cerebrovascular diseases as it would ease hemodynamic blood flow modeling (Chaplain *et al.,* 2006; Grassly and Fraser, 2008; Stergiopulos, 2011; Hirsch *et al.,* 2012). Data on tapering as presented could be of importance for pulse wave propagation modeling. The major sites for pulse wave reflection are thought to be at branching points and taper of arteries and arterioles. By confirming the lack of tapering the current data indicate that including tapering in modeling is not necessary (Hamilton, 1944; Nichols *et al.,* 2011). Besides branching patterns, the current study presents data on whether the cerebral arterial tree complies with the optimality principles as described by Murray in the law of minimum work. These optimality principles in turn are used in models like tissue metabolism driven arterial tree generation (Schneider *et al.,* 2012).

### Main Findings of the Study

A large dataset containing bifurcations and segments of the human cerebral arterial tree was created, enabling quantification of branching patterns.

#### Asymmetry‐ratio

There is a broad range in AS for all four methods. This shows that in the cerebral circulation bifurcations of various symmetries are present. Further there is a striking difference in AS in all four methods. With the lowest median AS of 0.09 for dissection and the largest of 0.78 for 9.4 T MRI. This difference might be explained by the number of included smaller side branches. The cerebral circulation is known to have many small side branches bifurcating in a sharp angle from the main arteries (Marinkovic *et al.,* 1985; Umansky *et al.,* 1985). With a voxel resolution of 0.1 mm for 7 T MRI, the maximum radius of a measurable artery is 0.1 mm, hence, these smaller side branches are not detected with the 7 T MRI technique. The same principle applies for the 9.4 T MRI. The manual cast measurements also show a higher AS (0.44) than the measurements on the dissected vessel tree. This may be the result of the finding that the smaller side branches of the cast are too small to measure by hand without breaking them.

Hence, AS in the current study is probably more a reflection of the amount of included smaller side branches. The AS of the dissection data might reflect the actual AS of the cerebral circulation as we believe the smaller side branches were lost.

#### Area‐ratio

The current study shows a large range in AR with an increasing AR when smaller arteries, closer to the microvasculature, are included. This matches well with results from literature.

Based on the principle of minimum work the AR would range between 1.10 for turbulent flow in larger arteries and 1.26 for laminar flow in smaller arteries (Uylings, 1977). From literature we know that in the human vasculature the range of AR is much larger than this theoretical range (Hutchins *et al.,* 1976; Hardy‐Stashin *et al.,* 1980; Fanucci *et al.,* 1990; Papageorgiou *et al.,* 1990; Aharinejad *et al.,* 1998; Beare *et al.,* 2011).

The wide range in AR found in the current study (between a median 1.05 and 1.45) might be partially explained by the difference in included side branches as reflected in the AS. Inclusion of small side branches will lead to a decrease in AR as those small branches only slightly increase the cross‐sectional area at those. As MRI data included less small side branches an increased AR can be expected when compared to manual measurements on dissected arterial trees. Additionally, it is expected when getting closer to the capillary circulation there will be less smaller side branches which will further increase AR. The 9.4 T MRI data included arteries close to the arteriole network and showed the highest AR of 1.45. This increasing trend in AR when including smaller arteries matches well with an even higher AR of 1.52 as shown by Cassot *et al*. (2010) who measured the cerebral microcirculation (mean ± SD diameter 6.91 ± 3.85 μm) in 9,414 bifurcations in India ink‐injected human brains.

Other studies show that the larger arteries of the cerebral circulation have indeed tendency of a lower AR. Rossitti and Löfgren (1993) studied 176 bifurcations on angiogram data with an AR of 1.2 ± 0.4. They included only arteries greater than 1 mm in diameter. Ingebrigtsen *et al*. (2004)) performed measurements on 69 3D angiograms of the larger bifurcations of the ICA, basilar artery (BA), and MCA for which the mean ± SD AR were, respectively, 0.8 ± 0.2, 0.7 ± 0.4, and 1.2 ± 0.3.

#### Principle of minimum work

The data of the current study, combined with the already existing data in literature (Suwa *et al.,* 1963; Wang *et al.,* 1992; Rossitti and Löfgren, 1993; Rossitti and Frisen, 1994; Ingebrigtsen *et al.,* 2004; Cassot *et al.,* 2009; Mut *et al.,* 2014) indicate that there is a large spread between different parts of the circulation and possibly between different subjects in regard to the principle of minimum work. However, when including most side branches the cerebral circulation seems to follow the principle of minimum work.

Traditionally studies calculate the exponent of the equation $r_{0}^{x}=r_{1}^{x}+r_{2}^{x}$ for minimum work as defined by Murray. Theoretically the exponent would be 2.33 in case of fully turbulent flow and three in case of fully laminar flow (Uylings, 1977; Pollanen, 1992). However, solving *x* in this equation is only possible by numerical methods and can result in values of plus or minus infinity as discussed by Sherman *et al*. (1989) Hence, in the current article, we chose the alternative by performing a simple regression analysis of the ratio between the cubed parent artery radius and the sum of the cubed daughter artery radii. The closer the slope to a value of one is, the more the arterial tree the principle of minimum work for laminar flow follows. The slopes as shown in Figure 4 were 1.13 ± 0.02, 1.36 ± 0.02, 0.90 ± 0.01, 1.04 ± 0.01, for 7 T, 9.4 T, manual cast, and dissection, respectively.

Again, for the principle of minimum work the inclusion of small side branches, as reflected in the AS, might influence this ratio. For the dissection data, which most likely included most side branches, the slope was almost equal to 1 with a very good fit of the regression analysis (*R* ^2^ = 0.96). This might confirm that the cerebral circulation follows the principle of minimum work.

Only a few other studies performed a comparable regression analysis to confirm the principle of minimum work as done in the current study. In two studies of human cerebral (Rossitti and Löfgren, 1993) and retinal (Rossitti and Frisen, 1994) arteries linear regression analysis demonstrated slopes of 0.87 ± 0.07 (mean ± SD) and 0.98. In a histological study by Wang *et al*. (1992) on 150 mice brain (366 bifurcations) the linear fit did not differ significantly from 1, showing that the mice cerebral circulation follows the principle of minimum work.

Most studies that calculated the exponent also demonstrate that the cerebral circulation shows a large range but follows the principle of minimum work. Suwa *et al*. (1963) showed for arteries greater than 0.1 mm in two human brains an exponent of 2.67 and 2.79.

Ingebrigtsen *et al*. (2004) analyzed 3D angiograms of 69 human patients and found an exponent of 1.7 ± 0.8, 1.2 ± 0.3, and 2.9 ± 1.2 for the major bifurcations of the ICA, basilar artery and MCA, respectively. In a study by Mut *et al*. (2014) which analyzed MRI's (voxel size 0.6 × 0.6 × 0.6 mm) of 61 human brains and found an exponent of 2.5. Cassot *et al*. (2009) showed an exponent of median 3.58 (IQR 2.29–6.14, range 0.662–1,947, 9,414 bifurcations) for the cerebral microvasculature. Rossitti and Löfgren (1993), who analyzed 12 angiograms of 10 adult patients, found an exponent of 2.9 ± 0.7 (for 157 bifurcations) for the ICA, ACA, and MCA for arteries larger than 1 mm in diameter.

#### Length‐radius‐ratio

LR was only acquired by dissection and both MRI techniques, as measurements of length on cast by manual techniques are not accurately possible. LR values for dissection and 7 T MRI data ranged from values close to 0 to almost 43. 9.4 T MRI showed LR values of 0 to almost 18. Median values were 6.82, 4.24, and 10.49 for 7 T MRI 9.4 T MRI and dissection, respectively. These values found in the current study were lower than those described in literature, but showed a comparable range. As with the other branching patterns LR might also be depending on the inclusion of small side branches. However, in mathematical flow models the length of the segments is of less influence on flow, as radius has the greatest impact on resistance for flow.

In literature Iberall (1967) estimated that for the cardiovascular system a good LR would be 50 ± 10. Koike *et al*. (1986) measured a LR of 9 on resin casts of the arterial tree of six dog lungs. Based on data of Cassot *et al*. (2006) and Lauwers *et al*. (2008) the LR for the human cerebral microvasculature would lie between 10.1 and 16.4 depending on whether median or mean values are used for calculation. Rai *et al*. (2013) measured the length and diameter of 100 human patients using CTA of the proximal cavernous ICA, the ICA terminus, the MCA origin and the M2 origin returning an LR of 13.4 and 13.1 for men and women, respectively, for ICA, and for MCA 15.3 and 14.1 for men and women. Finally, in a scanning electron microscopy study of capillaries of rat brain, lengths and diameters were published from which an LR of 30.5 (range 2.1–53.2) could be calculated (Motti *et al.,* 1986).

#### Tapering

Tapering showed median values of 0.99 for dissection and both MRI techniques, indicating that there is no significant tapering. However, tapering, just as the other branching patterns, showed a large range with values between 0.55–1.43 0.70–1.27 and 0.71–1.27, for 7 T MRI 9.4 T MRI and dissection, respectively.

To our knowledge, there are no quantitative data on tapering of the cerebral arterial tree in literature. Based on the principle of minimal work one would expect that tapering is not present. This is confirmed by the current data.

### Limitations

This study has several limitations: (a) for the different methods samples of different subjects were used. The differences in branching patterns found among the techniques in the current study might be caused by the usage of samples of different subjects. Since it is not known if the differences found are solely due to an intersubject variability, due to difference in technique or as suggested above, due to the difference in included smaller side branches. (b) A second limitation is the change in vascular muscle tone postmortem. It is known that postmortem the arterial smooth muscle relaxation and contractility change (Patel and Janicki, 1970; Onoue *et al.,* 1993). This change could have an impact on cast production and radius measurements performed on dissection material. (c) The method used to store the dissected arteries could have influence on the diameters and lengths measured. In literature there are indications that tissue, when fixed using formalin, results in shrinkage of tissue which might impact measurements performed in the current study (Su *et al.,* 2006; Kerns *et al.,* 2008). OMO laundry detergent could show a comparable effect, but this is unknown. (d) Another limitation mainly having effect on length measurements in dissection is the loss of axial stretch. When removing arteries using dissection their respective length is shortened by axial elasticity. To perform length measurements in dissection the arteries were stretched until straight, which would likely have caused inaccuracy in the length measurements. (e) For measuring the radius on dissected arteries both the width of a compressed artery and its arterial wall are measured. From these the radius is calculated. This method might introduce additional systematic error. (f) Although, there were no neuropathological defects visible on inspection of the brains used, age of the subjects might have had an effect on the morphology and branching patterns of the cerebral arterial tree. (g) Lastly, there was a difference in pressure at which the Araldite F mixture was injected in the casts used for manual measurement and MRI scanning. For the manual measurement cast pressure was controlled at a static pressure of 93 mm Hg until solidification was complete. However, for the MRI, casts from a previous unpublished study were used, in which pressure was of less importance as only complete penetration of the cerebral circulation up to the level of the capillaries was required. This could have resulted in a difference in pressure during production, which might impact the radius of the arteries. However as shown in a study by Guo *et al*. (2012) the inner radius of arteries show less increase in radius at increasing pressures. We expect the required pressure to result in adequate Araldite‐F penetration to be around the level at which the increase in radius with increasing pressure is only minimal. Hence, diameters should not differ much between the casts for manual cast measurements and casts used for MRI measurements due to possible difference in pressure during production of the casts.

It is, however, questionable if these limitations have a major and significant effect on the results and conclusions.

In conclusion, to our knowledge this is the first study to show data of branching patterns in this range (0.1–1.0 mm diameter) of arteries of the human cerebral arterial tree. The current data on branching patterns show, as other authors have done previously in the cerebral microvasculature (Cassot *et al.,* 2010), such a large spread that these branching patterns can only be interpreted as general trends. It is of importance that models take this large spread and variation throughout the cerebral arterial tree into account. Finally, the current work shows indications that on a macroscopic scale the cerebral arterial tree follows the principle of minimum work as described by Murray (1926b) and that the reduction of radius of cerebral arteries occurs predominantly at the level of bifurcations rather than along segments between bifurcations.
